# Verbal fluency functional magnetic resonance imaging detects anti‐seizure effects and affective side effects of perampanel in people with focal epilepsy

**DOI:** 10.1111/epi.17493

**Published:** 2023-01-08

**Authors:** Fenglai Xiao, Lorenzo Caciagli, Britta Wandschneider, Marine Fleury, Lawrence Binding, Davide Giampiccolo, Andrea Hill, Marian Galovic, Jaqueline Foong, Dong Zhou, Josemir W. Sander, John S. Duncan, Matthias J. Koepp

**Affiliations:** ^1^ Department of Clinical & Experimental Epilepsy UCL Queen Square Institute of Neurology London UK; ^2^ Chalfont Centre for Epilepsy Bucks UK; ^3^ Department of Neurology West China Hospital of Sichuan University Chengdu China; ^4^ Department of Bioengineering University of Pennsylvania Philadelphia Pennsylvania USA; ^5^ Department of Neurology The Royal London Hospital London UK; ^6^ Department of Neurology, Clinical Neuroscience Center University Hospital Zurich Zurich Switzerland; ^7^ Stichting Epilepsie Instellingen Nederland ‐ (SEIN) Heemstede The Netherlands

**Keywords:** anti‐seizure medication, fMRI, focal epilepsy, longitudinal, perampanel, undesired effects

## Abstract

Perampanel, a noncompetitive antagonist of the postsynaptic a‐amino‐3‐hydroxy‐5‐methyl‐4‐isoxazolepropionic (AMPA) receptor, is effective for controlling focal to bilateral tonic–clonic seizures but is also known to increase feelings of anger. Using statistical parametric mapping–derived measures of activation and task‐modulated functional connectivity (psychophysiologic interaction), we investigated 14 people with focal epilepsy who had verbal fluency functional magnetic resonance imaging (fMRI) twice, before and after the add‐on treatment of perampanel. For comparison, we included 28 people with epilepsy, propensity‐matched for clinical characteristics, who had two scans but no change in anti‐seizure medication (ASM) regimen in‐between. After commencing perampanel, individuals had higher task‐related activations in left orbitofrontal cortex (OFC), fewer task‐related activations in the subcortical regions including the left thalamus and left caudate, and lower task‐related thalamocaudate and caudate‐subtantial nigra connectivity. Decreased task‐related connectivity is observed between the left OFC and precuneus and left medial frontal lobe. Our results highlight the brain regions associated with the beneficiary therapeutic effects on focal to bilateral tonic–clonic seizures (thalamus and caudate) but also the undesired affective side effects of perampanel with increased anger and aggression (OFC).

## 1 INTRODUCTION

Perampanel, a non‐competitive antagonist of the postsynaptic a‐amino‐3‐hydroxy‐5‐methyl‐4‐isoxazolepropionic (AMPA) receptor, is licensed as an add‐on medication for people with focal seizures, with or without focal to bilateral tonic–clonic seizures (FBTCS).^1^ Feelings of anger or aggression are recognized side effects of perampanel.^2^

Verbal fluency functional magnetic resonance imaging (fMRI) has been used to investigate the neural correlates of FBTCS in temporal lobe epilepsy.^3^ Prior work showed altered activation and connectivity profiles of the thalamus and basal ganglia in people with FBTCS compared to those with focal seizures without secondary generalization, and of the orbitofrontal cortices of people with mood disturbances.^4^, ^5^, ^6^ Here, we used verbal fluency fMRI to assess changes in cognition‐related brain activity after introducing perampanel in people with epilepsy. We hypothesized that changes of activity in basal ganglia, thalamus, and frontal areas may be associated with the desired and undesired effects of perampanel.

## 2 METHODS

### 2.1 Participants

We analyzed routinely acquired fMRI data of adults with focal epilepsy, who had language fMRI scans as part of their presurgical evaluation at the National Hospital for Neurology and Neurosurgery, London, between January 2010 and March 2020. We identified people who had two task fMRI scans. We excluded people with excessive motion or failure to perform the tasks. People with brain lesions other than hippocampal sclerosis were excluded.^7^ Lateralization and localization of the epileptic focus was confirmed by experienced epileptologists, based on review of clinical, neurophysiological, and MRI or positron emission tomography (PET) and ictal electroencephalography (EEG) tracings during video‐EEG telemetry or ambulatory EEG monitoring. Ambiguous lateralization or localization was classified as undetermined.

To define a comparable “patient‐control” group with no anti‐seizure medication (ASM) changes between the two verbal fluency fMRI scans, we used propensity‐score matching in SPSS 26, with a ratio of 1:2 for the variables of age at scan, age at onset, sex, handedness, total number of medications, localization, and laterality of seizure focus.

The study was classified by the institutional review board as a service evaluation involving further anonymized analysis of previously acquired data that did not require individual participant consent.

### 2.2 MRI data acquisition, fMRI paradigm, and pre‐processing

Gradient echo‐planar images providing blood oxygen level–dependent (BOLD) contrast were acquired on a 3 T Excite HDx scanner (General Electric), using a standard 8‐channel receive coil. Each volume comprised 50 contiguous oblique axial slices, ensuring full brain coverage, with 2.5‐mm slice thickness, 64 × 64 matrix, and 24‐cm field of view, providing an in‐plane voxel size of 3.75 × 3.75 mm. Echo time was 25 milliseconds and repetition time was 2.5 seconds. During the paradigm, 30‐second task blocks were alternated with 30‐second blocks of crosshair fixation as a control condition. Participants were instructed to covertly generate words starting with a visually presented letter (A, D, E, S, W).

### 2.3 fMRI data analysis

Functional MRI data were preprocessed with Statistical Parametric Mapping 12 (SPM12), and underwent realignment, spatial normalization to scanner‐specific template in Montreal Neurological Institute (MNI) space, resampling (isotropic 3 × 3 × 3 voxels), and spatial smoothing with a Gaussian kernel of 8 mm full width at half‐maximum. At the first level, the task was modeled by convolving the vector of block onsets with a canonical hemodynamic response function to create regressors of interest; six motion parameters were included as confounds. Contrast images for each participant were created for task‐relevant activation and deactivation.

We entered activation contrasts for each individual into a full factorial design with group as a factor ([“pre‐perampanel”, “post‐perampanel”, “non‐antiseizure medication (ASM) change 1st scan”, and “non‐ASM change 2nd scan”]), and envisioned the following analyses: (1) one‐way analysis of variance (ANOVA) comparing groups “pre‐perampanel”, “post‐perampanel”, “non‐ASM change 1st scan”, and “non‐ASM change 2nd scan” with a 2 × 2 analysis to investigate the effect of perampanel; (2) two paired *t* tests comparing “pre‐perampanel” versus “post‐perampanel”; “non‐ASM change 1st scan” versus “non‐ASM change 2nd scan”. For a whole‐brain exploratory analysis, we set the threshold for statistical significance at *p* < .005 (uncorrected) with a cluster extent threshold of 20 contiguous voxels, to balance between type I and type II errors.^8^

We then used a psychophysiological interaction (PPI) analysis to test task‐related functional connectivity between activated areas. Individual fMRI time‐series were obtained from the preprocessed images using an 8‐mm radius sphere centered on individual, participant‐specific peak activation voxels in the left thalamus, left caudate, and left orbitofrontal cortex (OFC). The PPI general linear model included three regressors^1^: main effect of the seed region (i.e., the functional time series),^2^ task regressor (i.e., psychological factor, represented by the vector of the word‐generation block onset), and^3^ interaction of the former items, representing a task modulated change in connectivity, or PPI.^3^ Motion parameters were included as regressors of no interests. One‐sample *t* tests identified areas exhibiting task‐related connectivity changes with the seeds. Main PPI effects were thresholded at *p* < .05; family‐wise error (FWE) corrected across the whole brain. Pre‐ and post‐perampanel changes were compared by using paired *t* tests. Given prior work showing relevance of thalamus‐basal ganglia in FBTCS and frontal regions related to mood disorders,^4^, ^5^, ^6^ group differences were considered significant at *p* < .05. few corrected within a region of interest (ROI) consisting of a 12‐mm‐diameter sphere (small volume correction [FWE‐svc]) centered at the location of the maxima for thalamus and basal ganglia related to FBTCS and frontal regions related to mood disorders. For completeness, whole‐brain effects are reported at an exploratory statistical threshold of *p* < .005, k = 20.^7^, ^8^

### 2.4 Statistical analysis

Categorical variables are displayed as numbers and percentages, and they were analyzed with Fisher's exact test. Kruskal‐Wallis tests were employed for all other data. Analyses were conducted in SPSS 26 (IBM).

## 3 RESULTS

Of 1283 people with refractory focal epilepsy who underwent language fMRI during the above‐specified 10‐year period, 78 individuals had at least two verbal fluency fMRI scans. Of those 78 individuals, 14 people had received add‐on perampanel as the only ASM change between two consecutive scans, and 28 comparable subjects had no ASM changes between scans (Table 1).

**TABLE 1:** Demographic features between add‐on perampanel and no‐change groups

	Perampanel (*n* = 14)	No‐change (*n* = 28)	*p* Value
Sex, F/M	6/8	14/14	.662
Age at first scan, median (range), y	34.6 (21–46)	35.1 (23–46)	.762
Interval between scans, median (range), y	2.6 (0.7–6.9)	1.8 (0.8–6.9)	.196
Interval between add‐on PER and second scan, mean, (SD), mo	13.1 (8.2)	N/A	
Age at epilepsy onset, median, (range), y	18.0 (0.9–40.0)	16.0 (0.9–39.0)	.354
Handedness, right/left/ambidextrous	12/1/1	21/5/2	.642
Localization of epilepsy, temporal/frontal/parietal/undetermined	7/3/2/2	15/6/3/4	.989
Lateralization of epilepsy, left/right/undetermined	7/7/0	14/13/1	.769
Hippocampal sclerosis, *n* (%)	1 (7.1)	5 (17.1)	.350
Focal cortical dysplasia, *n* (%)	2 (14.3)	5(17.1)	.770
Seizure frequency at baseline, *n* (%)			.483
Monthly to weekly	5 (35.7)	12 (42.9)	
Weekly to daily	3 (21.4)	9 (32.1)	
Daily seizures	6 (42.9)	7 (25.0)	
Active FBCTS before the first scan, *n* (%)	5 (35.7)	14 (50)	.381
Abatement or decrease of FBCTS between first and second scan, *n* (%)	5 (35.7)	0 (0)	.000
Change of seizure frequency between the first and second scan, no change/decrease/increase	6/7/1	22/3/3	.019
Subjective feelings of aggression or anger after add‐on PER, *n* (%)	4 (28.5)	0 (0)	.009
HADS‐ Anxiety, mean (SD)	*n* = 11, 8.3 (5.3)	*n* = 26, 7.5 (4.6)	.803
HADS‐ Depression, mean (SD)	*n* = 11, 5.0 (4.1)	*n* = 26, 5.3 (4.0)	.714
Letter fluency, mean (SD)	*n* = 12, 12.3 (5.4)	*n* = 27, 11.5 (6.0)	.828
Animals fluency, mean (SD)	*n* = 12, 15.7 (5.7)	*n* = 27, 15.8 (5.8)	.863
Number of ASMs at baseline, mono/dual/triple/four	4/6/3/1	5/16/6/1	.774

Abbreviations: ASM, anti‐seizure medication; FBCTS, focal to bilateral clonic–tonic seizures; HADS, Hamilton anxiety and depression inventory; m, month; *n*, number; PER, perampanel; SD, standard deviation; y, year.

After a titration period, all 14 individuals were on a stable dosage of 8–10 mg/day at the time of the second scan. Six of the 14 individuals (42.8%) reported a decrease in overall seizure frequency (>50% decrease in 6 months), with five of those six individuals experiencing either a substantial decrease in FBTCS frequency (>50% decrease in 6 months) or no further FBTCS since commencing perampanel. One person (7.1%) reported an increase in seizure frequency.

None of the 14 individuals had a history of psychiatric illnesses. Four individuals (28.6%) reported subjective feelings of anger or irritability between two scans, that is, prior to the second scan. Anger issues were self‐reported and corroborated by carers or family members. No individual discontinued perampanel due to side effects.

Of those 28 people without changes in ASM between the two fMRI scans, 3 reported a decrease (>50% decrease in 6 months) and 3 an increase in seizures. Except for 3 of the 28 people who had a history of depression but were not taking antidepressant medication at the time of scanning, none had a history of any psychiatric illness. None of the 28 individuals self‐reported new‐onset feelings of anger or depression between the two scans.

**FIGURE 1:**
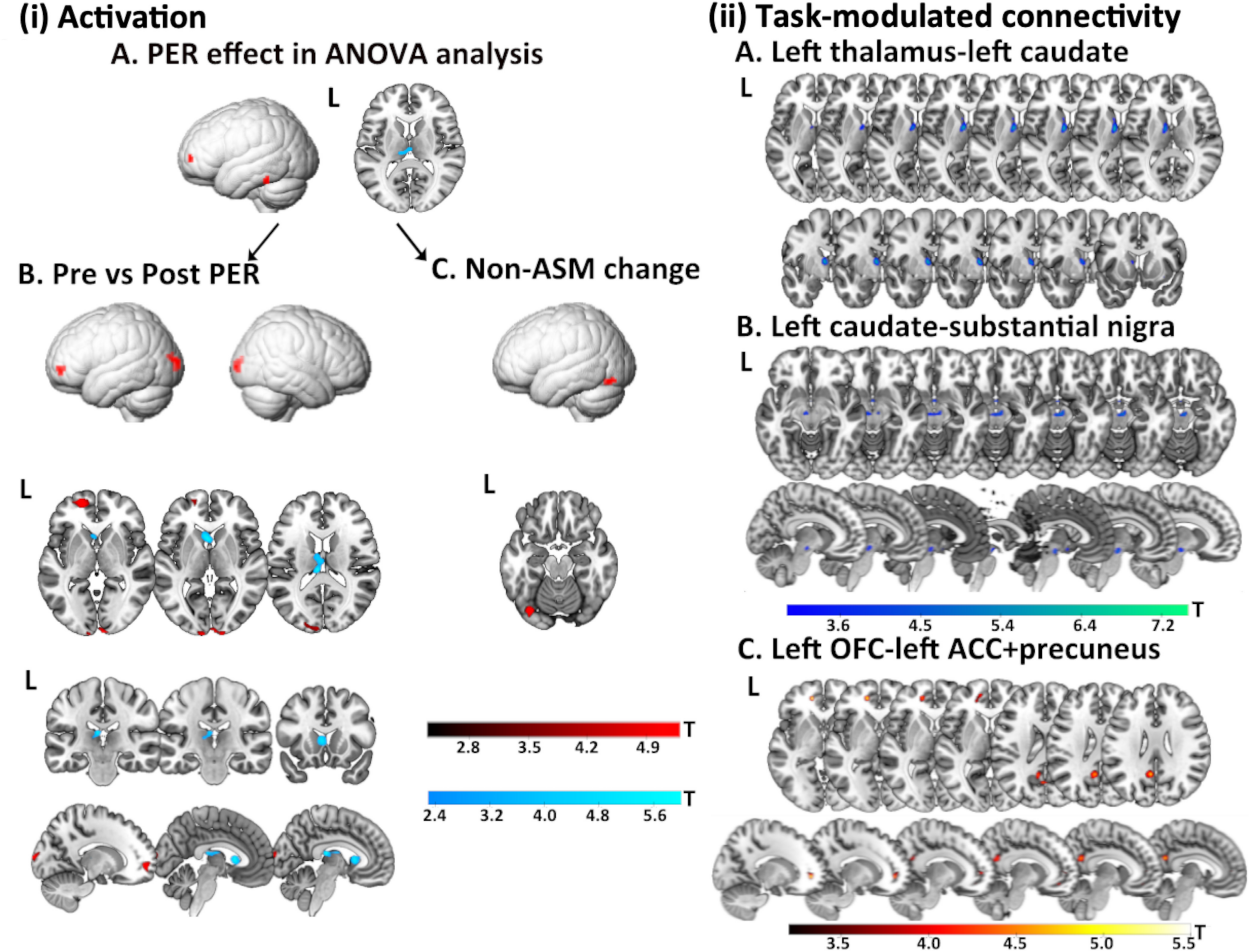
Changes in verbal fluency fMRI activation patterns before and after treatment with perampanel. (i) The whole‐brain verbal fluency activation: Cortical changes are demonstrated on a surface‐rendered brain template and subcortical changes are demonstrated superimposed on MNI 152 template (*p* < .001, k = 20), with a bar chart providing a *T* score scale with red bar indicating increased activation and cool bar decreased activation. (A) one‐way analysis of variance (ANOVA) among pre‐ and post‐treatment of perampanel, 1st scan of anti‐seizure medications (ASM) non‐change group and 2nd scan of ASM non‐change group (B) Paired *t* test of pre‐ and post‐treatment of perampanel. (C) Paired *t* test between the two scans of individuals in the group with no ASM changes. (ii) Psychophysiological interaction (PPI) analysis: cortical changes are demonstrated on a surface‐rendered brain template and subcortical changes are demonstrated superimposed on MNI 152 template (*p* < .005, k = 10), with a bar chart providing a *T* score scale. (A) left thalamus seed; (B) left caudate seed; (C) left OFC seed. ASM, antiseizure medication; L, left; OFC, orbitofrontal cortex; PER, perampanel; R, right.

Whole‐brain voxel‐wise ANOVA revealed significant post‐treatment effects of perampanel within the left OFC and left inferior temporal lobe (*p* < .001, k > 20). At a lower threshold (*p* < .01, k > 20), there were significant post‐treatment effects in the left thalamus (Figure 1, Table S1).

Comparing pre‐ and post‐treatment scans revealed: (1) decreased activation in the left caudate and thalamus, and (2) increased activation in the left OFC and medial occipital lobe (*p* < .001, k > 20) (Figure 1, Table S1). PPI analysis showed decreased task‐modulated connectivity between the left thalamic seed and left caudate, and between the left caudate seed and the substantia nigra (SNr) (*p* < .05, k > 20, FWE‐svc). A further decrease in connectivity was observed between the left OFC seed and left precuneus (*p* < .001, k > 20), and left anterior cingulate cortex (ACC) (*p* < .05, k > 20, FWE‐svc) (Figure 1, Table S1).

## 4 DISCUSSION

After commencing perampanel, people with focal epilepsy showed lower activation in the left thalamus and left caudate, and overactivity of the left OFC. Task‐related functional connectivity is lower between the thalamus and basal ganglia but increased between the left OFC and default mode regions. Compared to our “patient‐control” group, the add‐on perampanel group had fewer FBCTS with four individuals reporting new feelings of anger since starting perampanel.

We interpret the reduced activation in the left thalamus and caudate, coupled with weakened task‐related connectivity between caudate and SNr, as the fMRI correlate of improved seizure control on perampanel. Emerging evidence underscores the critical role of the thalamus and basal ganglia in shaping susceptibility to FBTCS.^9^, ^10^ Our recent verbal fluency fMRI study in focal epilepsy identified increased task‐related thalamic functional connectivity in those with FBTCS.^3^ Reduced seizure frequency in response to deep brain stimulation of basal ganglia and thalamus was documented for refractory focal epilepsy.^11^ Moreover, evidence from several experimental models of seizures and epilepsy indicates that increased inhibition of nigral neurons, achieved by locally administering γ‐aminobutyric acid (GABA)ergic drugs into the SNr, was responsible for a reduction or suppression of experimentally triggered seizures.^12^ The lower activation and weakened functional connectivity may be attributed to the action of perampanel on thalamic‐basal ganglia circuitry, which is reflected by better control of FBTCS in individuals after adding this drug.

The overactive left OFC, as well as higher task‐related connectivity with the precuneus and left ACC after perampanel may be related to the established side‐effect of angry feelings. Notably, four individuals self‐reported feelings of anger after taking perampanel, whereas none reported mood disorders or feelings of anger in the patient control group between the two scanning time points. Converging evidence from neuroimaging studies suggests that impulsive aggression is regulated by frontolimbic brain structures including the OFC and ACC.^13^ The OFC plays a key role in processing of anger and behavioral impulsivity.^14^ The subjective feeling of anger was associated with higher regional cerebral blood flow in the left OFC in healthy people.^15^ A meta‐analysis of 65 neuroimaging investigations found that activations for negatively‐valenced emotions were left‐lateralized in the OFC.^16^ Heightened functional connectivity between the left OFC and ACC predicted the extent of violent behavior reported in patients with schizophrenia,^17^ whereas stronger functional connectivity between left OFC and precuneus was negatively correlated with lower psychological resilience in the normal brain.^18^ Thus we hypothesize that the left OFC is likely to be hyperexcitable with higher doses of perampanel. Such an effect may not be captured at rest but becomes apparent when the brain is challenged by cognitive tasks such as verbal fluency, which recruit areas more markedly affected by medication changes.

Although the longitudinal design controls for most epilepsy‐related effects other than effects due to changes in ASMs, our study is limited by its retrospective nature and lack of randomization and is open to selection bias.

In summary, our longitudinal study delineates the effects of perampanel on the activity of thalamus, basal ganglia, and left OFC during language fMRI, and shows altered functional connectivity among FBTCS‐related subcortical regions and increased OFC connectivity that may relate to mood disturbances. These changes may represent the potential neurobiological substrates of the observed therapeutic effects on FBCTS and of the undesired, affective side effects of perampanel. A prospective longitudinal study is needed to confirm these findings.

## AUTHOR CONTRIBUTIONS

FX and MJK conceptualized and designed the study. FX, LC, and AH carried out the acquisition and processing of imaging data. FX, LC, BW, DG, LB, MF, and MG contributed to the imaging and statistical data analysis. FX interpreted the data and drafted the manuscript. JD, JWS, and MK supervised the data analysis, interpretation, and manuscript preparation. All co‐authors contributed to data interpretation and manuscript preparation. DZ and MK obtained funding. All authors had full access to all of the data (including statistical reports and tables) in the study and can take responsibility for the integrity of the data and the data analysis accuracy. All authors approved the final version of the manuscript before submission.

## CONFLICT OF INTEREST

The authors declare that this work was conducted in the absence of any commercial or financial relationships that could be construed as a potential conflict of interest. JWS has received personal fees from Eisai, UCB, GW Pharma, Arvelle, and Zogenix and research grants from UCB and GW Pharmaceuticals outside the submitted work. MJK has received grants and honoraria from UCB Pharma, Desitin, Novartis, Eisai, GE, and Bial, outside the submitted work. MG has received honoraria from Bial pharmaceutical and Nestlé Health Science outside the submitted work.

## Supporting information


Table S1.
