# Supplementary material for: Verbal fluency functional magnetic resonance imaging detects anti‐seizure effects and affective side effects of perampanel in people with focal epilepsy
Source: Epilepsia. 2023 Jan 8;64(2):e9–e15. doi: 10.1111/epi.17493 (PMC10107311; doi:10.1111/epi.17493)
Supplement: Supplementary file 1 — Table S1. [file EPI-64-e9-s001.docx]

**Supplementary Table .** Pretreatment to Posttreatment change in brain activation

| Verbal fluency activations | MNI coordinates (x, y, z) | Z score | P value |
| --- | --- | --- | --- |
| ANOVA, effects of perampanel |  |  |  |
| Left orbitofrontal cortex | -18, 62, 4 | 3.51 | <0.001 |
| Left inferior temporal lobe | -45, -43, 26 | 3.19 | 0.001 |
| Left Thalamus | -12, -25, 10 | 2.86 | 0.003 |
| Pre > Post Perampanel |  |  |  |
| Left caudate | 0, 11, 7 | 4.82 | <0.001 |
| Left Thalamus | -6, -25, 16 | 5.17 | <0.001 |
| Pre < Post Perampanel |  |  |  |
| Left orbitofrontal cortex | -24, 59, -5 | 4.08 | <0.001 |
| Middle occipital lobe | -5, -106, 10 | 4.33 | <0.001 |
| Non AED change |  |  |  |
| Left fusiform gyrus | 24, -49,67 | 3.40 | <0.001 |
| Functional connectivity analysis (PPI) |  |  |  |
| Decrease: left thalamus-left caudate | -9, -4, 10 | 4.34 (4.34*) | <0.001(<0.001*) |
| Decrease: left caudate- substantial nigra | 3, -16, -14 | 3.03 (3.03*) | 0.001(0.020*) |
| Increase: left orbitofrontal cortex-precuneus | -6, -55, 25 | 3.32 (3.32*) | <0.001 |
| Increase: left orbitofrontal cortex-left ACC | -15, 47, -2 | 3.66 (3.63*) | <0.001 (0.005*) |

Coordinates are given in MNI space. MNI, Montreal Neurological Institute. A scanner upgrade occurred in 2013, but the scanning protocol remained unchanged. * Statistically significant p values (p < 0.05, familywise error [FWE]-svc corrected) for peak-level differences. For task-related functional connectivity, p values were FWE-corrected for multiple comparisons using a 12-mm diameter priori hypothesis spherical region of interest (svc) centered on the local maximum. Other p values are uncorrected for multiple comparisons across the whole brain.
